# Conscription of Immune Cells by Light‐Activatable Silencing NK‐Derived Exosome (LASNEO) for Synergetic Tumor Eradication

**DOI:** 10.1002/advs.202201135

**Published:** 2022-06-04

**Authors:** Mengjie Zhang, Wanxuan Shao, Tongren Yang, Houli Liu, Shuai Guo, Deyao Zhao, Yuhua Weng, Xing‐Jie Liang, Yuanyu Huang

**Affiliations:** ^1^ School of Life Science Advanced Research Institute of Multidisciplinary Science School of Medical Technology (Institute of Engineering Medicine) Key Laboratory of Molecular Medicine and Biotherapy Key Laboratory of Medical Molecule Science and Pharmaceutics Engineering Beijing Institute of Technology Beijing 100081 China; ^2^ Department of Radiation Oncology the First Affiliated Hospital of Zhengzhou University Erqi Zhengzhou 450000 China; ^3^ Chinese Academy of Sciences (CAS) Center for Excellence in Nanoscience CAS Key Laboratory for Biomedical Effects of Nanomaterials and Nanosafety National Center for Nanoscience and Technology Beijing 100190 China

**Keywords:** exosome, immunotherapy, natural killer cells, reactive oxygen species, small interfering RNA

## Abstract

Exosomes derived from natural killer (NK) cells (NEO) constitute promising antineoplastic nano‐biologics because of their versatile functions in immune regulation. However, a significant augment of their immunomodulatory capability is an essential need to achieve clinically meaningful treatment outcomes. Light‐activatable silencing NK‐derived exosomes (LASNEO) are orchestrated by engineering the NEO with hydrophilic small interfering RNA (siRNA) and hydrophobic photosensitizer Ce6. Profiling of genes involved in apoptosis pathway with Western blot and RNA‐seq in cells receiving NEO treatment reveals that NEO elicits effective NK cell‐like cytotoxicity toward tumor cells. Meanwhile, reactive oxygen species (ROS) generation upon laser irradiation not only triggers substantial photodynamic therapy effect but also boosts M1 tumor‐associated macrophages polarization and DC maturation in the tumor microenvironment (TME). In addition, ROS also accelerates the cellular entry and endosomal escape of siRNA in TME. Finally, siRNAs targeting PLK1 or PD‐L1 induce robust gene silencing in cancer cells, and downregulation of PD‐L1 restores the immunological surveillance of T cells in TME. Therefore, the proposed LASNEO exhibit excellent antitumor effects by conscripting multiple types of immune cells. Considering that its manufacture is quite simple and controllable, LASNEO show compelling potential for clinical translational application.

## Graphical abstract

Light‐activatable silencing natural killer (NK)‐derived exosome (LASNEO) enables robust immuno‐oncotherapy by conscripting multiple types of immune cells. It elicits inherited NK cell‐like cytotoxicity. Reactive oxygen species photogeneration triggers effective photodynamic therapy, boosts M1 macrophage polarization and DC maturation, and facilitates endosomal escape of small interfering RNA. PD‐L1 silencing and soluble factors further restore T cell immune surveillance.

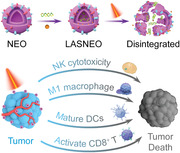

## 1 Introduction

Natural Killer (NK) cells are lymphocytes in the same family as T and B cells, coming from a common progenitor. As an important effector in innate immunity, NK cells exhibit a significant anti‐tumor effect and possess a broad range of applications in tumor immunotherapy.^[^ ^1^ ^]^ There are several mechanisms by which NK cells induce apoptosis of stressed cancer cells:^[^ ^1b^ ^]^ i) Several tumor necrosis factor (TNF) superfamily members such as Fas ligand (FasL) and TNF‐related apoptosis‐inducing ligand (TRAIL) expressed by NK cells induce apoptosis of target cells via binding to their corresponding receptors FAS or TRAILR, respectively. ii) Cytotoxic proteins, including perforin, granzymes, and other lytic granule molecules released by NK cells, induce cell death in stressed cells; iii) NK cells mediate antibody‐dependent cell‐mediated cytotoxicity upon engagement of CD16 (Fc*γ*RIIIA) by target cells coated with antibodies. iv) NK cells secrete various inflammatory cytokines (e.g., interferon *γ* (IFN‐*γ*), TNF‐*α*, interleukin‐10 (IL‐10)), growth factors (granulocyte‐macrophage colony‐stimulating factor (GM‐CSF)) and chemokines (e.g., C‐C motif chemokine ligand 3 (CCL3), C‐C motif chemokine ligand 4 (CCL4), C‐C motif chemokine ligand 5 (CCL5), X‐C motif chemokine ligand 1 (XCL1)), which recruit and activate other immune cells such as T cells, dendritic cells (DCs), and macrophages.

Because NK cells exert sophisticated functions, they and their derivative biological agents represent attractive choices for developing immune modulators. However, additional orchestration of NK cells to enhance their killing capability on the targeted cells is required to achieve potential clinical translation application. Introducing a chimeric antigen receptor (CAR) to NK cells to prepare CAR‐NK cells constitutes a feasible strategy investigated in clinical trials.^[^ ^2^ ^]^ In addition, exosomes are nano‐sized vesicles actively secreted by many different cells and are usually responsible for intercellular communication and cargo transfer.^[^ ^3^ ^]^ Exosomes containing proteins associated with cells, such as cytokines and growth factors thus possess similar functions to that of derived parental cells.^[^ ^4^ ^]^ For example, more recently, mesenchymal stem cell (MSC) derived exosomes are being examined for their role in MSC‐based cellular therapy.^[^ ^5^ ^]^ Meanwhile, exosomes (30–120 nm) are typically much easier to traffic to and penetrate solid tumors than the parent cells (≈10 µm).^[^ ^6^ ^]^ Therefore, exosomes derived from NK cells have also been deemed an appealing therapeutic agent in tumor immunotherapy because they maintain the essential immune‐stimulatory ability of NK cells.^[^ ^7^ ^]^ Several studies have demonstrated that exosomes derived from NK cells possess specific tumor‐cell‐killing properties and lack cytotoxic activity against normal cells.^[^ ^8^ ^]^ It was reported that NK‐derived exosomes carrying the tumor suppressor microRNA (miR)‐186 exhibited cytotoxicity against neuroblastoma and inhabited immune escape.^[^ ^9^ ^]^ However, significantly enhancing the killing effects of NK‐derived exosomes (NEO) is a fundamental and challenging issue for their in‐depth applications in cancer treatment.

Re‐education of immune cells in the solid tumor microenvironment (TME) is a deliberate strategy to combat cancer.^[^ ^10^ ^]^ Oncotherapy by blocking immune checkpoints has attracted widespread attention. Programmed cell death 1 (PD1) and PD1 ligand 1 (PD‐L1) is an important immune checkpoint mediating the suppression of the immune system. PD‐1/PD‐L1 interaction modulation by employing antibodies,^[^ ^11^ ^]^ small molecules,^[^ ^12^ ^]^ and small interfering RNAs (siRNAs)^[^ ^13^ ^]^ has proven to be an effective treatment strategy. Among them, siRNA can meditate target mRNA cleavage in a sequence‐specific manner, which supports inhibition of endogenous expression of the target gene,^[^ ^14^ ^]^ while antibodies or small molecules can only block the interaction of the expressed PD‐1/PD‐L1. However, establishing an effective and safe delivery system remains a challenging but meaningful mission for developing siRNA‐based cancer treatment modality.^[^ ^13^, ^15^ ^]^

Furthermore, reactive oxygen species (ROS) play a major role in immune regulation, differentiation, and functions, which are necessary for developing the immune response.^[^ ^16^ ^]^ ROS generation by photosensitizer in TME, endosome/lysosome, or cytoplasm plays a vital role in macrophages reprogramming^[^ ^17^ ^]^ and drug release.^[^ ^15c^ ^]^ In addition, ROS‐involved photodynamic therapy (PDT) is an attractive clinically approved treatment modality for cancer therapy comprising photosensitive drugs and laser activation.^[^ ^18^ ^]^ Irradiating the tumor site with specific wavelength activates photosensitizers that selectively cluster in the tumor tissue, triggering a photochemical reaction that destroys tumor tissue.^[^ ^19^ ^]^

**Scheme 1:**
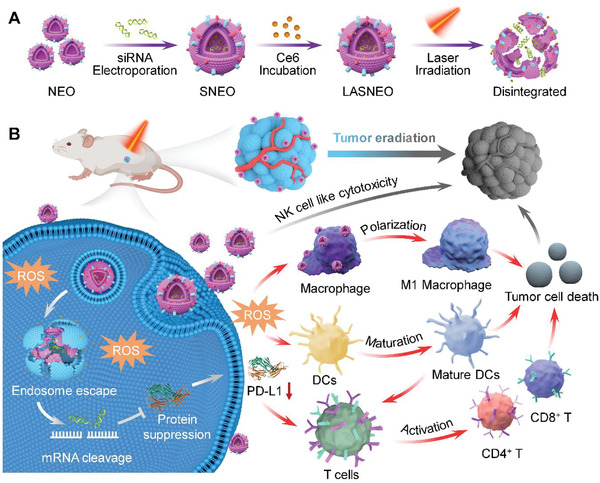
Schematic illustration of LASNEO mediated synergetic tumor eradication. A) Fabrication of siRNA and Ce6 dual‐loaded LASNEO and the light‐triggered disassemble of LASNEO. B) Collaboratively reprogram of multiple types of immune cells by LASNEO. First, LASNEO displays NK cell like cytotoxicity. After internalized by tumor cells, LASNEOs are disintegrated under 660 nm laser irradiation and photogenerated ROS facilitates endosomal escape of siRNA, and then siRNA mediates robust gene silence of PLK1 or PD‐L1. Downregulation of PD‐L1 and several soluble factors contained in NEO restore T cell immune surveillance. Moreover, ROS also triggers effective photodynamic therapy and augments M1 macrophage polarization and DC maturation.

Therefore, we developed a light‐activatable silencing NK‐derived exosome (LASNEO) system (**Scheme** **1**). LASNEO was simply prepared by electroporating hydrophilic siRNA into the NK cells‐derived exosomes (NEO) and then incubating with hydrophobic photosensitizer of Chlorin e6 (Ce6) (Scheme 1A). After administration, LASNEO can induce tumor cells death directly via introducing NK cell‐like cytotoxicity in tumor tissue. Meanwhile, after internalized by tumor cells, ROS will be generated upon laser irradiation at 660 nm, which not only achieves an effective PDT, but also facilitates reprogramming macrophages to M1 phenotype and maturing dendritic cells (DCs) in TME. In addition, ROS will also destroy the endolysosomal membrane and facilitate the release of siRNA from the exosomes and endosomes/lysosomes. The siRNA then triggers robust gene silencing of polo like kinase 1 (PLK1) or PD‐L1. The inhibition of PD‐L1 activates CD4+ T cells and CD8+ T cells in TME. Hence, the proposed LASNEO shows promising prospects in cancer treatment because it conscripts multiple types of immune cells to kill the pathological cell. It also holds great clinical translation potential as its manufacturing process is quite simple and controllable.

## 2 Results and Discussion

### 2.1 Characterization and Immunocompetence of NEO

**Figure 1:**
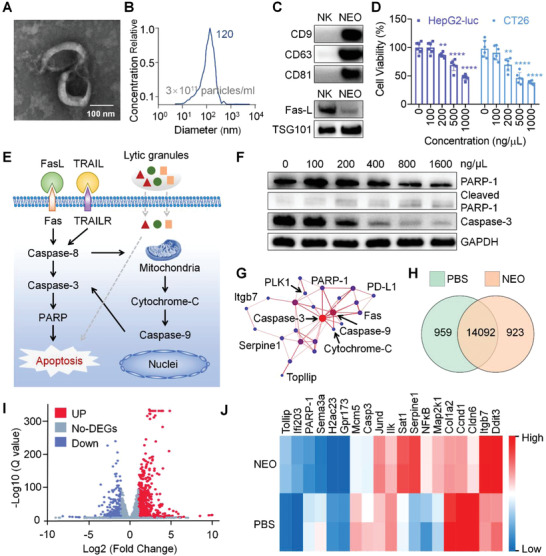
Comprehensive characterization of NK‐derived exosome (NEO). A) Transmission electron microscopes (TEM) image of NEO. Scale bar, 100 nm. B) Particle size distribution of NEO recorded by NTA. C) Western blot analysis of CD9, CD63, CD81, Fas‐L, TSG101 in NEO. D) Cytotoxicity of NEO evaluated in HepG2‐Luc and CT26 cells. **p < 0.01, ****p < 0.0001. E) Proposed regulation pathways of NEO toward cancer cells. FasL, Fas ligand; TRAIL, TNF‐related apoptosis‐inducing ligand; PARP1, poly (ADP‐ribose) polymerase 1. Representative lytic granules include perforin, granzymes, granulysin, etc. F) Western blot analysis of crucial proteins involved in apoptosis pathway. The concentrations of NEO were 0, 100, 200, 400, 800, 1600 ng µL^−1^, respectively. G) Protein−protein interaction networks, as determined from RNA‐seq data. H) A Venn diagram revealed the number of genes transcribed in PBS and NEO treated CT26 cells. I) Volcano plots displayed the up expressed (red) and down expressed (blue) genes. Genes that were not differentially expressed were denoted as no‐DEGs. J) A heat map of gene transcriptions of interest.

NK‐92MI cells were cultured in the exosome‐free medium for 24 h. NK cells derived exosomes (NEO) were then isolated from the culture supernatant by differential centrifugation.^[^ ^20^ ^]^ According to the guidance released by the International Society for Extracellular Vesicles in 2018,^[^ ^21^ ^]^ exosome identification methods, including transmission electron microscope (TEM), nanoparticle tracking analysis (NTA) (NanoSight Range, Malvern Panalytical), and Western blots were employed to characterize NEO, respectively. The TEM imaging clearly showed that NEO were uniformly spherical, with a distinct membrane structure and size of ≈120 nm (**Figure** **1**A). The specific number and particle size distribution based on NTA revealed that NEO were physically homogenous particles with a peak around 120 nm in diameter and a concentration around 3 × 10^11^ particles mL^−1^ (Figure 1B and Figure S1, Supporting Information), which were consistent with the results of TEM. Moreover, Western blot results suggested that the common markers of exosomes (CD9, CD63, CD81, Tumor Susceptibility Gene 101 (TSG101)) and FasL were displayed on NEO (Figure 1C). These data proved that NEO with high quality and purity were successfully prepared.

The cytotoxicity of the NK cells toward tumor cells was confirmed first. It was observed that NK92MI cells triggered significant cell death of HepG2‐Luc and CT26 cells, respectively (Figure S2A,B, Supporting Information). Encouraged by these results, we performed MTT assay on tumor cells to determine the cytotoxicity of NEO. Different doses of NEO were incubated with HepG2‐Luc and CT26 cells for 24 h. Data showed that NEO also significantly decreased the viability of both tumor cells in a dose‐dependent manner (Figure 1D). In addition, to further confirm the specific killing effect of NEO, we also evaluated the viability of HEK‐293 cells and human umbilical vein endothelial cells (HUVEC) after treating with NEO at different concentrations (ng µL^−1^). No cytotoxicity was observed in these two normal cell types (Figure S3A,B, Supporting Information). Meanwhile, HEK‐293‐derived‐exosome also exhibited no cytotoxicity toward HepG2 and CT‐26 cells (Figure S4A,B, Supporting Information).

### 2.2 Immune Regulation Mechanism of NEO

According to the well‐defined working mechanism of NK cells toward the target cells, the assumed regulation pathway mediated by NEO is shown in Figure 1E. In brief, several transmembrane proteins, such as FasL and TRAIL, induce caspase‐dependent apoptosis via binding to their corresponding receptors of FAS and TRAILR (respectively) on the target cell surface.^[^ ^1^, ^7^, ^22^ ^]^ Meanwhile, lytic granules such as perforin, granzymes B, and granulysin inherently contained in NEO inducing target cell apoptosis. To confirm if NEO inherited the cytotoxicity regulation capability from NK cells, Western blot and RNA sequencing (RNA‐seq) was performed after validating the tumor cell‐killing ability of NEO on CT26 and HepG2‐Luc. Here, CT26 cells were incubated with NEO for 24 h and then the expression levels of PARP‐1, cleaved PARP‐1, caspase‐3, cleaved caspase 3, caspase 9, cleaved caspase 9, and cytochrome‐C proteins in CT26 cell were examined by Western blot. The results showed that PARP‐1, caspase‐3, and caspase 9 were significantly downregulated in a dose‐dependent manner (Figure 1F, Figure S5, Supporting Information). Accordingly, the expression of cleaved PARP‐1, cleaved caspase 3, and cleaved caspase 9 was upregulated (Figure 1F, Figure S5, Supporting Information). These results suggested that FasL and TRAIL displayed on NEO bound to FAS and TRAILR, respectively, activating caspase‐8 and downstream apoptosis pathways. Meanwhile, after treatment with NEO, the activated caspase‐8 in the cytoplasm enhanced the permeability of the mitochondrial membrane, and then the contents in the mitochondrial, including cytochrome‐C were released into the cytoplasm (Figure S6, Supporting Information).

In addition to interaction with target cancer cells on the cell membrane and secretion of lytic granules to cancer cells, the cellular uptake of NEO by cancer cells (HepG2‐Luc) was also examined by Confocal laser scanning microscopy (CLSM). Here, NEO was stained with lipophilic green fluorescent dye 3,3′‐dioctadecyloxacarbocyanine perchlorate (DiO), and the Confocal images suggested that NEO was effectively internalized by the cells and localized around the nuclei (Figure S7, Supporting Information), suggesting that lytic granules might also trigger apoptosis directly in the cytoplasm by taking the boat of NEO. These studies indicated that NEO triggered cancer cell death via the NK cell‐like apoptosis pathway.

To further confirm this mechanism, whole‐transcriptome analysis with total RNA‐seq was performed on NEO‐treated CT26 cells. The protein−protein interaction in apoptotic pathways and Kyoto Encyclopedia of Genes and Genomes (KEGG) network diagram were shown in Figure 1G and Figure S8, Supporting Information, respectively, revealing that NEO initiated a similar cell apoptosis pathway with NK cells. The transcripts of a total of 15 974 genes were examined. 923 genes were transcribed in the NEO‐treated cells (Figure 1H). Compared with the PBS‐treated cells, 699 genes were upregulated (red dots), and 359 genes were downregulated (blue dots) in the NEO‐treated cells (Figure 1I). Through KEGG enrichment analysis, different genes were divided into five branches: cellular processes, environmental information, processing genetic information, processing metabolism, and organismal systems (Figure S9, Supporting Information). Subsequently, we analyzed all interest genes in different pathways. Heat map clustering manifested those genes such as caspase‐3 and PARP‐1 were downregulated (Figure 1J), in line with the results of corresponding protein expression (Figure 1F). Overall, Western blot and RNA‐seq data provided a comprehensive understanding of the changes in protein and transcriptome expression and the alterations in cytotoxic pathways.

### 2.3 Fabrication and In Vitro Characterization of LASNEO

**Figure 2:**
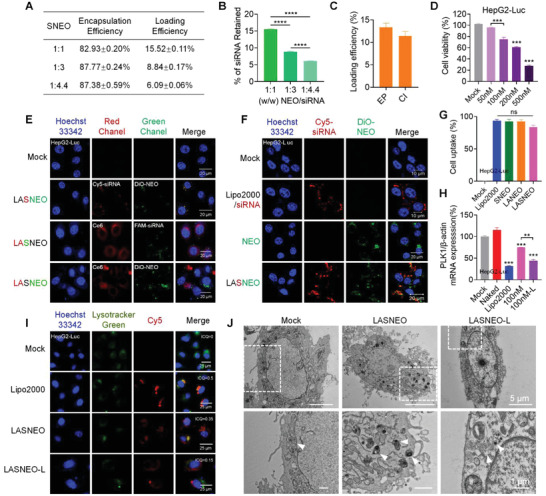
Preparation and in vitro performances of LASNEO. A,B) Determination of the encapsulation and loading efficiencies of siRNA. C) Determination of Ce6 loading efficiency in the samples prepared by electroporation (EP) or co‐incubation (CI). D) The inherited NK cell‐like cytotoxicity of LASNEO toward HepG2‐Luc cells. The cells were treated with 660 nm laser irradiation at 0.1 W cm^−2^ for 2 min. E) Observation of the transfection of LASNEO in HepG2‐Luc cells by confocal laser scanning microscopy (CLSM). Scale bars: 20 µm. Cy5 (red) or FAM (green) labeled siRNA was used in this assay. NEO was stained with DiO (green). The fluorescence signal was detected in different samples and channels. F) Confocal images analysis cellular uptake of NEO and LASNEO in HepG2‐Luc cells. Cell nuclei, siRNA, and NEO were counterstained with DAPI (blue), Cy5 (red), DiO (green), respectively. G) FACS analysis of cellular uptake of Lipo2000/FAM‐siRNA, SNEO, LANEO, and LASNEO. Quantitative results of Lipo2000/FAM‐siRNA, SNEO, and LASNEO were determined by FAM signal, and that of LANEO was determined by Ce6 signal, respectively. The assays in (D), (E), (F), and (G) were repeated in triplicate. H) Relative expression of PLK1 mRNA in HepG2‐Luc cells. Cells were treated with LASNEO carrying anti‐PLK1 siRNA, with or without laser irradiation. I) Confocal observations of siRNA distribution in HepG2‐Luc cells treated with LASNEO, with or without irradiation at 4 h after transfection. siRNA was labeled with Cy5 (red). Endosome/lysosome and nuclei were stained with Lysotracker Green (green) and Hoechst 33342 (blue), respectively. Intensity correlation quotient (ICQ) analysis was performed to determine the colocalization ratio of red and green pixels. Scale bar, 25 µm. J) BioTEM analysis of CT26 cells receiving the treatments of LASNEO and laser irradiation (0.1 W cm^−2^, 2 min). As indicated by the white dotted lines, the images displayed in the lower panel were enlarged from the areas of the upper panel images. The white arrowheads indicated LASNEO in endosome and the damaged endosome membrane of CT26 cells after treatment with laser irradiation. Scale bars: 5 µm (upper panel) and 1 µm (lower panel), respectively. **p < 0.01; ***p < 0.001; ****p < 0.0001; ns, not significant difference.

Hydrophilic siRNA was loaded into NEO by electroporation to prepare to silence NK‐derived exosome (SNEO). We evaluated the loading efficiency of siRNA at different mass ratios of NEO and siRNA (1:1, 1:3, 1:4.4, w/w), suggesting that the loading efficiency reached the highest value (≈16%) at the mass ratios of 1:1 (**Figure** **2**A,B). In addition, we verified the gene silencing of SNEO in HepG2‐Luc. It was observed that SNEO triggered ≈35% gene silencing at a mass ratio of 1:1 (at siRNA concentration of 100 nm) (Figure S10, Supporting Information). These data proved that siRNA has been successfully encapsulated by NEO and could be transfected to target cells by NEO.

In addition, hydrophobic Chlorin e6 (Ce6), a widely‐used photosensitizer, was employed to fabricate the LASNEO. The introduction of Ce6 confers LASNEO following regulation capabilities. First, upon light irradiation, Ce6 can effectively generate ROS, and mediate excellent PDT by interacting with cellular components such as amino acid residues, unsaturated lipids, and nucleic acids. Second, ROS can re‐educate the TME, including reprogramming macrophages to M1 phenotype and inducing DC maturation. Moreover, ROS generation can also accelerate the cellular entry and endosomal escape of siRNA in TME by oxidizing and destroying the membranes, a unique mechanism of photochemical internalization (PCI),^[^ ^15^, ^23^ ^]^ showing excellent synergistic effects with siRNA.^[^ ^15c^ ^]^ Because both exosome membrane and Ce6 intrinsically were hydrophobic, we tested both co‐incubation and electroporation methods to load Ce6 into SNEO.^[^ ^24^ ^]^ It was observed that there was no significant difference between these two methods, and the loading efficiency for incubation saturated at Ce6 concentration at 125 ng µL^−1^, with a loading efficiency of ≈13.37% (Figure 2C, and Figure S11, Supporting Information).

Consequently, the capability of LASNEO in killing cancer cell was evaluated in vitro. LASNEO was used to treat cancer cells at different siRNA (negative control siRNA, siNC) concentrations (50, 100, 200, 500 nm), and MTT recorded the cell viabilities after laser irradiation and 24 h incubation. The results showed LASNEO exhibited dose‐dependent cancer cell killing to both HepG2‐Luc (Figure 2D) and CT26 cells (Figure S12, Supporting Information).

### 2.4 Cellular Uptake of LASNEO

To further verify the successful fabrication of LASNEO and its ability in mediating cellular uptake, we stained the NEO with DiO, and labeled siRNA with Cy5 or FAM fluorophore. CLSM observations revealed that the NEO (in green) and siRNA (in red) were well co‐localized with each other in HepG2‐Luc cells, proving that the siRNA and Ce6 were successfully co‐loaded in NEO (Figure 2E, panel 2). In addition, subcellular observations of FAM‐labeled siRNA (in green), DiO‐stained NEO (in green), and Ce6 (in red) (Figure 2E, panels 3 and 4) demonstrated that LASNEO was effectively internalized by HepG2‐Luc cells.

Subsequently, we compared the transfection efficiencies of NEO, SNEO (siRNA‐loaded NEO), LANEO (Ce6‐loaded NEO) and LASNEO (Ce6 and siRNA dual‐loaded NEO), and commercial Lipofectamine 2000 (Lipo2000) by both Confocal imaging (Figure 2F) and fluorescence‐activated cell sorting (FACS) (Figure 2G, Figures S13 and S14A–C, Supporting Information) in HepG‐Luc cells. No significant difference was observed among these formulations regarding their cellular uptake performances. Lipo2000/siRNA and LASNEO showed comparable transfection efficiency, and functionalization with siRNA or Ce6 did not change the internalization behavior of NEO.

In addition, we also verified the cellular uptake of SNEO and LASNEO in CT26 cells. FACS data revealed that the uptake efficiencies of Lipo2000/FAM‐siRNA, SNEO (FAM‐siRNA), LASNEO (FAM‐siRNA) were overall comparable (Figure S14D, Supporting Information). The subcellular distribution of DiO‐stained NEO (in green) and Cy5‐siRNA (in red) was also recorded (Figure S15, Supporting Information), and then internalization of Ce6 (in red) and FAM‐siRNA (in green) was further examined (Figure S16, Supporting Information). Similar patterns with HepG2‐Luc cells were recorded, and LASNEO still displayed comparable transfections efficiency with Lipo2000.

More importantly, we continued to clarify whether macrophages would internalize LASNEO, which was a critical issue when LSAENO accumulated in TME. Accordingly, macrophages and CT26 cells were co‐cultured, and LASNEO were labeled with DiO. When sorting with FACS, macrophages were labeled with F4/80 antibody, and CT26 cells were labeled with SDCCAG3 antibody. It was revealed that most LASNEO was taken up by tumor cells (37.8%), and very few were internalized by macrophages (5.2%) (Figure S17, Supporting Information), demonstrating that tumor cells were the dominant target cell for the proposed formulation.

These data together elaborately demonstrated that 1) siRNA and Ce6 were successfully co‐loaded into the NEO, 2) LASNEO constituted a simple and effective carrier for delivering both hydrophilic agents (siRNA) and hydrophobic molecule (Ce6), 3) LASNEO was dominantly internalized by tumor cells.

### 2.5 Gene Silencing and Endosomal Escape of Internalized LASNEO

siRNA targeting Polo‐like kinase 1 (PLK1) (siPLK1) was used to investigate the gene silencing activity of LASNEO. After being treated with LASNEO for 4 h, HepG2‐Luc cells were washed with PBS and exposed to 0.1 W cm^−2^, 660 nm laser for 2 min. After incubation for another 20 h, total RNA was extracted and quantified with RT‐qPCR. It was observed that LASNEO (at siRNA concentration of 100 nm) achieved an ≈57.0% knockdown of PLK1 mRNA, which was comparable to the control of Lipo2000/siPLK1 (68.0% knockdown) (Figure 2H). It was worth noting that, compared to the silencing efficiency of LASNEO without laser treatment, the LASNEO with laser irradiation displayed higher gene silencing activity.

Rapid and efficient endosomal escape plays an essential role in siRNA delivery.^[^ ^13^, ^25^ ^]^ It was reported that ROS generated upon irradiation could interact with the lysosome/endosome membrane and facilitate siRNA escaping into the cytoplasm. It was assumed that ROS photogeneration in this study would disrupt both NEO and lysosome/endosome membrane, leading to effective siRNA escape and cytosolic release. To examine LASNEO‐assisted siRNA internalization and endosomal escape, Cy5‐labeled siRNA and lysotracker green were used to perform Confocal imaging. The colocalization ratio between siRNA and lysosome/endosome was calculated and employed as an index of endosomal escape efficiency. It was observed that upon irradiation with a 660 nm laser, the colocalization ratio significantly decreased from 0.35 to 0.15 (Figure 2I). In line with the above observations, a similar pattern was recorded in CT26 cells (Figure S18, Supporting Information). These results further suggested that ROS promotes the release of siRNA from lysosomes/endosomes into the cytoplasm.

In addition, Bio‐TEM was applied to directly observe the cellular uptake and endosomal release of LASNEO and the damage to cancer cells induced by LASNEO after laser irradiation. As shown in Figure 2J, LASNEO was successfully transfected into cells and first entrapped in lysosome/endosome. While after laser irradiation, the lysosome/endosome membrane was destroyed, resulting in siRNA escaping from the endosome/lysosome. Moreover, compared to the untreated control group (Mock), the cells underwent significant apoptosis after laser irradiation in LASNEO treated group. These results indicated Ce6‐produced ROS not only destabilized the membrane but also triggered cell apoptosis.

### 2.6 Effects of ROS Production on Macrophage Polarization and Tumor Cell Killing

**Figure 3:**
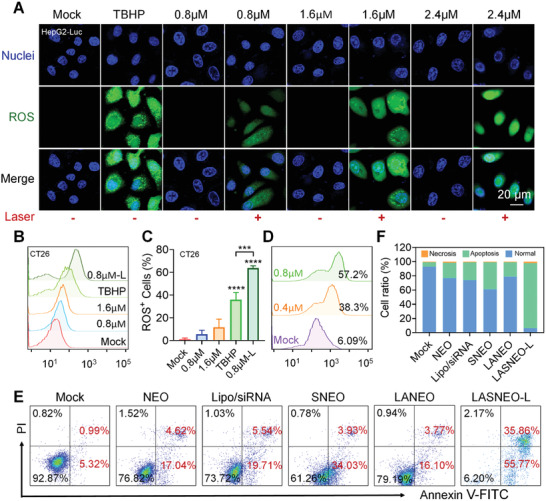
The photogeneration and biological effects of ROS in LASNEO‐treated cells. A) ROS levels in HepG2‐Luc cells after treating with LASNEO at different Ce6 concentrations (0.8, 1.6, and 2.4 µm). The ROS was stained with DCFH‐DA, and cells treated with ROS‐up solution were used as positive controls (TBHP). Scale bars, 20 µm. B) FACS determination of ROS production in CT26 cells. C) Quantitative analysis of (B) (*n* = 3). ***P < 0.001; ****P < 0.0001. D) FACS analysis of CD86 (M1 macrophage marker) expression on macrophages before and after treating with different concentrations of LASNEO and laser irradiation. E) FACS‐recorded apoptosis in CT26 cells after treating with LASNEO. The cells were stained with Annexin V‐FITC and PI. F) Quantitative analysis of (E). The assays in (A), (B), and (E) were repeated in triplicate.

CLSM and FACS confirmed ROS production. DCFH‐DA (2′, 7′‐dichlorodihydrofluorescein diacetate), a cell‐permeable fluorescent probe, was employed to detect intracellular ROS in HepG2‐Luc and CT26 cells. LASNEO at different Ce6 concentrations (0.8, 1.6, and 2.4 µm) were transfected into cells for 4 h. Then the cells were irradiated under a 660 nm laser for 2 min. Tert‐butyl hydroperoxide (TBHP) (100 µm) was employed as a positive control for ROS generation. After incubation for another 20 h, DCFH‐DA was added to the dish and reacted with cells for 1 h. The confocal imaging revealed that laser irradiation significantly enhances ROS production in a dose‐dependent manner (**Figure** **3**A). FACS data further confirmed the above observations (Figure 3B,C). Compared to the positive control group, laser irradiation caused a significant increase in ROS, as the ROS‐positive cell population increased from 36% to 63.8% after irradiation (Figure 3C).

More importantly, we further determined if LASNEO‐generated ROS involved in macrophage polarization. RAW264.7 cells were treated with IL‐4 for 48 h and then incubated with LASNEO for 4 h, the supernatant then was discarded, and the cells were irradiated with laser for 2 min. After further incubation for another 20 h, the expression of CD86, the marker protein of M1 macrophage, was analyzed by flow cytometry. Data revealed that the CD86 expression was increased in a dose‐dependent manner (Figure 3D). More RAW264.7 macrophage cells (M0 type) were polarized to M1 macrophage in the wake of LASNEO concentration increasing. This confirmed that Ce6‐induced ROS triggered robust cell apoptosis and proved that ROS reprogramed macrophages to the M1 phenotype.

To verify the PDT effect of LASNEO, flow cytometry analysis was performed to evaluate the cell apoptosis following LASNEO treatment. Annexin V‐FITC and Propidium Iodide were employed to identify the apoptotic state of cells (Figure 3E,F). In accordance with the MTT data (Figures 1D and 2D), the NEO treatment induced slight cell apoptosis (21.6% apoptosis cells versus control). At the same time, SNEO caused moderate cell apoptosis (37.96%) owing to the siRNA contribution. LASNEO with laser irradiation caused the highest apoptosis ratio of ≈ 91.5%, confirming the synergistic killing effect of LASNEO (Figure 3F).

### 2.7 In Vivo Anti‐Tumor Effect of LASNEO in HepG2‐Xenograft Liver Cancer Model

**Figure 4:**
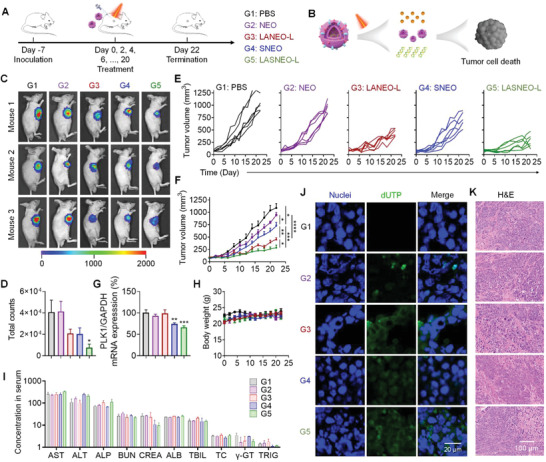
In vivo antitumor efficacy of LASNEO in HepG2‐Luc cell line xenograft tumor model. A) Treatment schedule and grouping information. Six animals were used in each group. B) Schematic illustration of the treatment principle. C) Bioluminescence imaging of the mice receiving different treatments. D) Quantitative analysis of all living mice receiving various treatments. E) Individual and average F) tumor volumes recorded during the treatment course. G) PLK1 mRNA expression in tumor tissues recorded at the end of experiment. H) Body weights recorded during the treatment course. I) Serum biochemistry parameters were analyzed at the end of treatment. Ten indicators were analyzed, including aspartate aminotransferase (AST, U L^−1^), alanine aminotransferase (ALT, U L^−1^), alkaline phosphatase (ALP, U L^−1^), blood urea nitrogen (BUN, mg dL^−1^), serum creatinine (CREA, µmol L^−1^), albumin (ALB, g L^−1^), total bilirubin (TBIL, µmol L^−1^), total cholesterol (TC, mmol L^−1^), *γ*‐glutamyl transpeptidase (*γ*‐GT, U L^−1^), Triglyceride (TRIG, mmol L^−1^). J) CLSM images of tumor cryosections prepared with tumors collected at the end of experiment. Sections were stained with DAPI and FITC‐labeled phalloidin. The scale bar was 20 µm. K) H&E staining of the tumor sections. The scale bar was 100 µm. *p < 0.05; **p < 0.01; ***p < 0.001; ****p < 0.0001.

Encouraged by the above observations, the in vivo therapeutic effects of LASNEO were further investigated by using the HepG2‐Luc‐xenograft murine model (**Figure** **4**A). When the tumor volumes reached around 100 mm^3^, mice were divided into 5 groups with 6 animals per group and treated with 1) PBS, 2) NEO, 3) LANEO‐L, 4) SNEO, and 5) LASNEO‐L, respectively, every other day. Suffix of “L” means laser irradiation was applied in corresponding groups of animals. siPLK1 was employed in this study because PLK1 is overexpressed in various human cancers and is associated with a poor cancer prognosis. Conceptually, after LASNEO accumulates in tumor tissue, NEO will induce tumor cell death through NK cell‐like regulation pathway (Scheme 1B, Figures 1E and 4B). ROS produced by Ce6 after laser irradiation and siRNA released from LASNEO will synergistically cause cell death via multiple regulation mechanisms, including PDT, immune cell conscription, and siRNA‐triggered apoptosis (Scheme 1B, Figures 1E and 4B).

Because HepG2‐Luc stably expresses firefly luciferase, in vivo bioluminescence imaging was performed at the end of the experiment. The intensity of the bioluminescence signal from the tumor tissue reflected the size of the tumor. Data showed that the tumor progression of the LASNEO‐L group (LASNEO with laser treatment) was significantly repressed (Figure 4C). The quantitative analysis of the fluorescence signal also confirmed these observations (Figure 4D). Tumor growth curves exhibited that the tumor progression was dramatically repressed in group of LASNEO‐L (Figure 4E,F). Meanwhile, LANEO‐L, SNEO, and NEO groups also showed satisfactory antineoplastic effects, and the treatment efficacy of the LANEO‐L group was second only to the LASNEO‐L group.

In addition, the expression of PLK1 mRNA was determined in tumor tissue at the end of the experiment. As a result, both SNEO and LASNEO‐L triggered gene silencing significantly, and LASNEO‐L exhibited higher gene inhibition efficiency than SNEO, which may be attributed to ROS generation facilitated cellular entry and endosomal escape in tumor cells (Figure 4G).

Moreover, the body weight was monitored during the whole treatment course (Figure 4H). Ten serum biochemistry parameters including aspartate aminotransferase (AST), alanine aminotransferase (ALT), alkaline phosphatase (ALP), albumin (ALB), total bilirubin (TBIL), *γ*‐glutamyl transpeptidase (*γ*‐GT), blood urea nitrogen (BUN), serum creatinine (CREA), total cholesterol (TC) and triglyceride (TRIG), were analyzed at the end of study (Figure 4I). No significant difference was observed for all these parameters compared to the corresponding values in the PBS group, suggesting the animals' well‐tolerated proposed formulations.

Finally, apoptosis in tumor tissues was examined at the end of the experiment. TUNEL staining of tumor sections showed significant cell apoptosis in all four treated groups (Figure 4J). LASNEO‐L group exhibited the highest level of positive fluorescence staining. Moreover, the H&E‐stained tumor sections also proved that the cells underwent pathological structural changes (Figure 4K), following TUNEL results. While no significant change was observed in other main organs, including the heart, liver, spleen, lung, and kidney (Figure S19, Supporting Information).

### 2.8 Immunotherapy of LASNEO in CT26‐Xenograft Tumor Model

**Figure 5:**
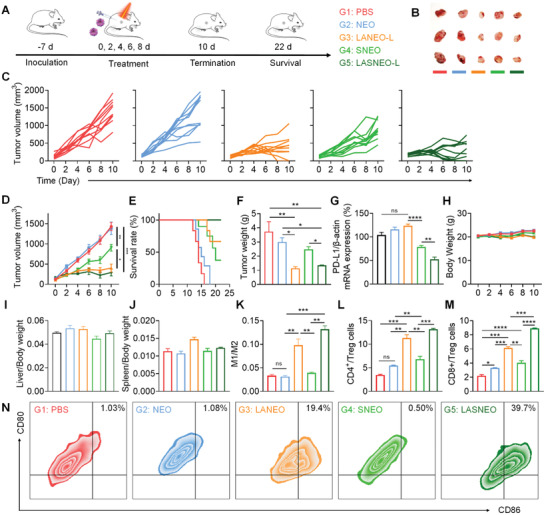
Immunotherapy of LASNEO evaluated with CT26‐xenograft murine tumor model. A) Treatment schedule and grouping information. Ten animals were used in each group. B) Optical images of three isolated tumors when the average tumor volume of PBS reached ≈2000 mm^3^. C–E) The tumor growth and survival curves of the animals during the treatment course. F) Tumor weight recorded at the end of experiment. G) Relative PD‐L1 mRNA expression in tumor tissues evaluated by RT‐qPCR. H) Body weights recorded during the treatment course. (I and J) Organ coefficients of the I) liver and the J) spleen. K) The ratios of M1 macrophages to M2 macrophages in tumor tissues. L,M) FACS‐recorded the radios of the CD8+ T cells/Tregs and CD4+ T cells/Tregs in tumors received various treatments. N) FACS determination of DC maturation in tumor tissues. CD11c+, CD80+ and CD86+ are the markers for matured DCs. *p < 0.05; **p < 0.01; ***p < 0.001; ****p < 0.0001; ns, not significant difference.

To further thoroughly investigate the immunotherapy effects of LASNEO, siRNA targeting programmed death‐ligand 1 (PD‐L1) (siPD‐L1) was employed. PD‐L1 and its receptor programmed death‐1 (PD‐1) are well‐defined regulators allowing some cells (e.g., cancer cells) to escape attack by the immune system.^[^ ^26^ ^]^ Plenty of therapeutic agents targeting the PD‐L1/PD‐1 pathway have achieved unprecedented treatment effects in combating various cancers. In this study, tumor model was established with a CT26 cell line that highly expressed PD‐L1^[^ ^27^ ^]^ (**Figure** **5**A). Five groups of animals received the treatment of PBS, NEO, LANEO‐L, SNEO, and LASNEO‐L, respectively. Suffix of “L” means laser irradiation was applied in corresponding groups of animals. The tumor growth was monitored during the whole treatment course. When the average tumor volume reached ≈1500 mm^3^ in PBS group, three animals in each group were randomly selected, and the tumor tissues were isolated and optically imaged. It was observed that the tumor size in the LASNEO‐L group and the LANEO‐L group was remarkably smaller than that in the PBS group (Figure 5B). The tumor growth curves suggested that the LASNEO‐L with laser irradiation exhibited excellent tumor growth inhibition (Figure 5C,D). Animals in LASNEO‐L group also showed longest survival duration (Figure 5E). Tumor weights recorded when the average tumor volume reached ≈1500 mm^3^ in PBS group revealed that LASNEO‐L and LANEO‐L dramatically inhibited tumor growth (Figure 5F).

Determination of the expression of PD‐L1 mRNA in tumor tissues demonstrated that both LASNEO‐L and SNEO triggered robust gene silencing, and the inhibition efficiency of LASNEO‐L was higher than SNEO, highlighting the contribution of irradiation‐induced ROS (Figure 5G). In addition, no significant change in body weight was observed during the whole treatment course (Figure 5H). The organ coefficients of the liver and spleen also remained in the normal range for all treatment groups (Figure 5I,J). Meanwhile, examination of ten serum biochemistry parameters (Figure S20, Supporting Information) and H&E‐stained tissue sections (Figure S21, Supporting Information) at the end of the experiment suggested that no remarkable difference could be observed among all five treatment groups. These data together proved that LASNEO‐L and all other tested formulations did not induce significant in vivo toxicity.

At last, we carefully explored the immune regulation mechanism of the proposed LASNEO. First, we isolated individual tumor cells through cell strainers, labeled the macrophages with antibodies, and analyzed the type of macrophages by flow cytometry. CD11b (integrin alpha‐M, ITGAM, integrin alpha‐X, ITGAX) is a 165 kDa adhesion molecule that expressed on the surface of macrophages and is one of the best markers for this population of cells. The CD11b positive cells are confirmed as macrophages. By using CD11b antibodies, we successfully separated macrophages from tumor tissues. Then, the macrophages were incubated with CD206 (marker protein of M2 macrophages) and CD86 (marker protein of M1 macrophages) antibodies and then analyzed by FACS. As a result, the population of M1 macrophages was significantly increased in both LANEO‐L and LASNEO‐L groups, which ascertained that the ROS promoted macrophages transited to M1 macrophages (Figure 5K). Second, we also analyzed CD3^+^ CD8^+^ T cells, CD3^+^ CD4^+^ T cells, and CD3^+^ Foxp3^+^ T cells (Treg cells) in the tumor tissues by FACS. Data suggested that the ratios of CD4^+^ T cells to Treg cells and CD8^+^ T cells to Treg cells were increased in LANEO‐L and LASNEO‐L groups (Figure 5L,M). Especially noteworthy was that the ratios of CD4^+^ T cells to Treg cells and CD8^+^ T cells to Treg cells were also elevated in groups of NEO and SNEO. It was reported that NK cells could produce some soluble factors, such as TNF‐*α*/*β*, IFN‐*γ* (interferon *γ*), GM‐CSF (granulocyte macrophage colony‐stimulating factor). It has been proved that TNF‐*α* and IFN‐*γ* were also contained in NEO.^[^ ^7^, ^28^ ^]^ This allows them to shape the immune response by interacting with immune cells, for example, T cells. Finally, the type of DC cells was determined with CD80 and CD86 antibodies, and CD11c was employed as the gate. The results indicated that the LANEO‐L and LASNEO‐L promoted the maturation of DC cells in TME (Figure 5N). Therefore, LASNEO achieved excellent onco‐therapy effects in murine tumor models by eliciting NK‐like cytotoxicity and re‐educating multiple types of immune cells, including M1 macrophage, mature DC, and cytotoxic T cells.

## 3 Conclusion

In a nutshell, we have successfully engineered a LASNEO that could synergistically conscript the immune cells in TME, enabling immuno‐oncotherapy effectively. First, NEO inherited the cytotoxicity property of NK cells and displayed effective killing ability toward cancer cells. Second, ROS generation under irradiation achieved effective PDT and promoted M1 macrophage polarization and DC maturation. Third, siPD‐L1 loaded in LASNEO and some soluble factors inherently contained in NEO restored the immunological surveillance function of CD4+ T cell and CD8+ T cell in TME. Finally, ROS also significantly facilitated the endosomal escape and cytosolic release of siRNA in targeted cells, enabling robust RNA interference (RNAi) therapy. Therefore, the proposed LASNEO re‐educated immune cells in TME through various mechanisms, showing desirable cancer therapeutic effects. This formulation is quite simple and effective and thus has broad clinical application prospects in tumor immunotherapy.

## 4 Experimental Section

### Materials and Reagents

Lipofectamine 2000, Dulbecco's modified Eagle's medium (DMEM), *α*‐minimal essential medium (*α*‐MEM), Opti‐MEM, fetal bovine serum, horse serum, penicillin‐streptomycin, 0.25% trypsin‐EDTA, DiO dye, Exosome Spin Columns, Micro BCA Protein Assay kit, as well as CD11b, CD86, CD206, CD11c, CD80 monoclonal antibodies were purchased from Thermo Fisher Scientific, USA. Calnexin, CD9, CD63 and CD81, FASL, Caspase‐3, Caspase‐8, and PARP antibodies were purchased from Abcam. 3‐(4, 5‐dimethylthiazol‐2‐yl)‐2,5‐diphenyltetrazolium bromide (MTT), RNA*later*, dimethyl sulfoxide (DMSO), LysoTracker Green DND‐26, isothiocyanate‐labeled phalloidin and Hoechst 33342 were purchased from Sigma Aldrich, USA. Filter membranes and ultrafilter tubes were purchased from Millipore (Shanghai, China). Cy5‐labeled siRNA (Cy5‐NC), siPLK1 (targeting PLK1), and siPD‐L1 (targeting PD‐L1) were supplied by Suzhou Ribo Life Science Co., Ltd. (Suzhou, China) or Suzhou Biosyntech Co., Ltd (Suzhou, China). All the primers were provided by BioSune Co., Ltd (Shanghai, China). DCFH‐DA, Annexin V‐FITC/PI Apoptosis Detection Kit, and Hieff qPCR SYBR Green Master Mix were purchased from Yeasen (Shanghai, China). Chlorin e6 (Ce6) was purchased from Macklin Biochemical Technology Co., Ltd (Shanghai, China). Optimal cutting temperature (OCT) compound was from Sakura Finetek USA, Inc. (Torracne, CA90501, USA).

### Cell Culture

NK‐92MI cells were obtained from the American Type Culture Collection (ATCC, Manassas, VA, USA) and cultivated in Alpha Minimum Essential medium without ribo‐nucleosides and deoxyribonucleosides but with 2 mm L‐glutamine and 1.5 g L^−1^ sodium bicarbonate. The medium was also supplemented with 12.5% (v/v) exosomes‐depleted fetal bovine serum, 12.5% (v/v) exosomes‐depleted horse serum, 1% (v/v) penicillin/streptomycin solution, 0.2 mm inositol, 0.02 mm folic acid and 0.1 mm 2‐mercaptoethanol. Exosome‐depleted serum was obtained after ultra‐centrifugation for 24 h, and the pellet was discarded. HepG2‐Luc, CT26, and RAW264.7 cells were obtained from the Type Culture Collection of the Chinese Academy of Sciences (Shanghai, China) and cultured in DMEM containing 10% (v/v) FBS and 1% (v/v) penicillin/streptomycin solution. All cell lines were cultured at 37 °C in a humidified atmosphere of 5% CO_2_.

### Isolation and Characterization of NEO

To obtain pure NEO, ultracentrifugation with cell culture supernatant was performed according to previously reported methods. In brief, NK‐92MI cells were cultured with an exosome‐free medium for 48 h, the supernatant was collected and centrifuged at 800 rpm for 10 min to remove NK‐92MI cells. The supernatant solution was then centrifuged at 2000 × *g* for 10 min and 10 000 × *g* for 30 min to remove dead cells and cell debris. The pellets were discarded after successive centrifugation, and supernatant was passed through a 0.22 µm sterilized filter membrane. After that, the filtrate was collected using pipettes and harvested by ultracentrifugation with a SW45Ti rotor (Beckman Coulter, USA) at 100 000 × *g* for 70 min at 4 °C. Then, NEO pellet was washed in cold PBS and the suspension was centrifuged at 100 000 × *g* for another 70 min for further purification. Subsequently, the NEO were re‐suspended in cold PBS and stored at −80 °C. To confirm the successful isolation of NEO, TEM and NTA were used for exosome quantification and characterization.

For TEM analysis, NEO was mixed with an equal volume of 4% paraformaldehyde (PFA) for 10 min. The mixture was added to sealing film, and formvar carbon film of copper grids was placed face down on the suspension for 20 min. The copper grids (Formvar film facing down) were washed with PBS droplets. Put the copper net on 1% glutaraldehyde drop for 5 min and wash it with PBS for three times. Then the copper net was put on the uranium oxalate droplet at pH 7 for 5 min. Finally, electron microscopic images were taken using Tecnai spirit TEM (FEI, USA) under 80 kV.

NTA was carried out by a ZETA VIEW device. After adjusting with PBS to a concentration of 1 mg of protein per milliliter solution, NEO were diluted with water and injected into the device system. The system focuses a laser beam through a suspension of the particles of interest. The Brownian motion of each particle was tracked between frames, ultimately allowing the calculation of the size through the application of the Stoke–Einstein equation.

Exosomes were also examined by dynamic light scattering analysis to evaluate the size distribution. Western blot was executed to detect marker proteins of exosomes. Antibodies against the following proteins were used: CD9, CD81, CD63, Calnexin, FasL, PARP‐1, cleaved‐PARP‐1, caspase‐3, and cytochrome‐C.

### Preparation of LASNEO

To load hydrophilic siRNA into NEO, negative control (NC) siRNA was used to optimize the electroporation procedure. NEO solution with 100 µg protein was diluted in PBS and mixed with different concentrations of siRNA to a total volume of 400 µL and incubated on ice for 10 min. Electroporation was carried out at 400 V and 125 µF in a 4 mm gap cuvette with Gene Pulser Xcell electroporator (Bio‐Red electroporation system). The cuvette electrode plates were made of aluminum, allowing for a uniform pulse delivery to the entire system. After electroporation, the mixture was pipetted gently to dissolve the aggregates formed during electroporation and incubated at 37 °C for 30 min to promote the fusion of the exosome membrane. SNEO (siRNA‐loaded NEO) was obtained after removed excess siRNA by ultracentrifugation. To quantify the efficiency of siRNA electroporation, the Ribogreen fluorescence was measured using Cytation5 microplate reader (BioTek, USA). All the experimental steps were completed under ice conditions. LASNEO was then prepared by co‐incubating Ce6 with SNEO. Briefly, Ce6 was dissolved in DMSO with 5 mg mL^−1^ concentration and mixed with SNEO at 37 °C for 1 h. The mixture was subjected to ultrafiltration at 3000 × *g* for 15 min to remove free Ce6, the purified LASNEO was obtained. Notably, LASNEO was freshly prepared and used in this study for both in vivo and in vitro experiments.

### Western Blotting

Western blotting was also performed to evaluate the purity of NEO or the apoptosis induced by NEO. At 24 h post‐transfection, protein was extracted with passive lysis buffer (50 mm tris, pH 7.4, 150 mm sodium chloride, 1 mm EDTA, 1% Triton X‐100). The cells or NEO lysate were centrifuged at 12 000 rpm for 10 min. Afterward, the protein was collected and protein concentration was measured by a BCA protein assay kit (CoWin Biosciences). 20 µg of protein lysates were separated on 10% polyacrylamide gels and transferred to a Nitrocellulose membrane (GE Healthcare). PageRuler Plus Prestained Protein Ladder (Thermo Scientific) was loaded on two sides of the samples. Membranes were blocked with 5% non‐fat milk in Tris‐buffered saline containing 0.1% Tween‐20 (TBST) for 1 h at room temperature and incubated with primary antibodies overnight at 4 °C. The membranes were washed three times with TBST and then incubated with HRP‐conjugated anti‐mouse and anti‐rabbit secondary antibodies for 1 h at room temperature. The blots were imaged using a 5200 multi‐automated chemiluminescence system (Tianneng, Shanghai, China). The intensity of the bands was quantified and subtracted by the background using ImageJ software.

### RNA‐Seq

CT26 cells were seeded in 6‐well plates (2 × 10^5^ cells well^−1^). After being incubated overnight, cells were treated with PBS and NEO in the culture medium at 37 °C, respectively. After 24 h post‐incubation, total RNA was extracted by TRIzol reagent. Dr. Tom was used for RNA‐seq analysis.

### Cell Viability and Apoptosis Assay

HepG2‐Luc or CT26 cells were cultured at 37 °C in a humidified atmosphere containing 5% CO_2_. HepG2‐Luc or CT26 cells were seeded in 96‐well plates at 1 × 10^4^ cells per well and cultured for 24 h. Then, the culture media was replaced by Opti‐MEM containing NEO at various mass concentrations (0, 100, 200, 500, 1000 ng µL^−1^) or LASNEO at different molar concentrations for siRNA (0, 50, 100, 200, 500 nm) and incubated for another 4 h. Then, after being washed with PBS, laser groups were exposed to 660 nm laser (0.1 W cm^−2^) for 2 min. The cells were cultured in fresh media for 20 h.

For cell viability analysis, the 3‐(4,5‐dimethyl‐2‐thiazolyl)‐2,5‐diphenyl‐2H‐tetrazolium bromide (MTT) assay was used to evaluate the cell viabilities of these cells. Briefly, MTT solution (10 µL, 5 mg mL^−1^) was added to each well and incubated with HepG2‐Luc (or CT26) cells for 4 h. After that, 200 µL DMSO was added to each well to dissolve formosan crystals. Absorbance was measured at an optical density of 562 nm in a spectrophotometric plate reader with a reference wavelength of 650 nm. Cell viability was calculated according to the following formula:

(1) $$\mathrm{Cell}\mathrm{viability}\left(\%\right)=\frac{OD_{540\left(\mathrm{Sample}\right)}-OD_{650\left(\mathrm{Sample}\right)}}{OD_{540\left(\mathrm{Mock}\right)}-OD_{650\left(\mathrm{Mock}\right)}}\times 100$$

OD_540_ represents the absorbance at 540 nm. OD_650_ represents the absorbance of 650 nm.

HepG2‐Luc and CT26 cells in 6‐well plate were treated with LASNEO as described above for apoptosis assay. After another 20 h incubation, cells were treated with Annexin V‐FITC Apoptosis Detection Kit to determine the apoptosis by flow cytometry analysis, according to the manufacturer's directions.

### Confocal Imaging

To verify the internalization and intracellular distribution of LASNEO in cells, HepG2‐Luc or CT26 cells were seeded in 6‐well (2 × 10^5^ per well) plates and cultured at 37 °C for 24 h. siRNA used here was labeled with Cy5 (in red) or FAM (in green). The exosome could be stained with 3,3′‐dioctadecyloxacarbocyanine perchlorate (DiO) (in green), and Ce6 loaded in LANEO or LASNEO could be excited with red light (in red). The cells were transfected with LASNEO or SNEO at a siRNA concentration of 100 nm. After being transfected for 4 h, the laser groups were washed with PBS, then exposed to 660 nm laser (0.1 W cm^−2^) for 2 min. After another 2 h incubation, cells were washed with PBS three times and observed using a confocal laser scanning microscope (CLSM) to determine cellular uptake and subcellular localization.

### Fluorescence‐Activated Cell Sorting (FACS) for Detection of Cell Uptake

To examine cellular uptake of LASNEO nanoparticles, 2 × 10^5^ HepG2‐Luc cells (or CT26 cells) were plated in 6‐well plates. Transfection was performed according to similar protocol to the Confocal assay. Cells were then digested with trypsin, washed with 1 mL 1 × PBS for three times, and suspended in 400 µL 1 × PBS. Finally, the fluorescence signal of Cy5 (or FAM, or DiO, or Ce6) was detected by a FACS Calibur flow cytometer (BD, USA). Mean fluorescence intensity was quantitatively analyzed by Flowjo 7.6.1. In detail, the cells were initially gated according to FSC‐A and SSC‐A to remove debris and dead cells. According to the width of FSC and the height of FSC, the cells were further gated to exclude doublets and aggregates. Subsequently, the fluorescent‐positive beads or cells were gated in the appropriate fluorescent channels: FITC for FAM or DiO, APC for Cy5 or Ce6, as the populations that exhibited negligible signals in the unstained/untreated negative controls.

### Confirmation of the Target Cell Type of LSANEO

To clarify, LASNEO were internalized by which types of cells, tumor cells, macrophages, or both. Macrophages and CT26 cells were co‐cultured and then transfected them with LASNEO labeled with DiO. The co‐cultured cells treated with 1) PBS; 2) LASNEO; 3) LASNEO and labeled with isotype control (F4/80 and SDCCAG3) antibody; 4) LASNEO loaded with anti‐PD‐L1 siRNA and labeled with F4/80 and SDCCAG3 antibody; 5) LASNEO loaded with siNC and labeled with F4/80 and SDCCAG3 antibody, respectively. Here, macrophages were labeled with F4/80 antibodies, and CT26 cells were labeled with SDCCAG3 antibodies before FACS. Then the FITC positive cells were first gated and the fluorescent signal of APC and PE‐Texas Red was recorded, respectively.

### RT‐qPCR Assay

HepG2‐Luc cells or CT26 cells were seeded in 6‐well plates (2 × 10^5^ cells well^−1^). After being incubated overnight, cells were treated with LASNEO at a siRNA concentration of 100 nm. Lipofectamine 2000 was used as a control. After incubation for 4 h, cells of all groups were washed with PBS and the laser groups were exposed to a 660 nm laser (0.1 W cm^−2^, 2 min). After 20 h post‐incubation, total RNA was extracted and retro‐transcribed with MultiScribe Reverse Transcriptase and oligo‐d(T) primers following total RNA purification with Trizol (Invitrogen), according to the manufacturer's protocol. Quantitative PCR (qPCR) analyses were performed on an ABI QuantStudio 3 using Hieff qPCR SYBR Green Master Mix. GAPDH or *β*‐actin was utilized as a housekeeping gene. Each reaction included three technical replicates, averaged to define one biological replicate. The experiments were repeated three times on distinct days, and each experiment defined a biological replicate. Statistical analyses were performed on Δ Ct of biological replicates (mice or independent experiments). The results were expressed as relative expression levels by normalizing to the negative control group.

### BioTEM

BioTEM was performed to directly observe the effects of irradiation‐induced ROS on membrane destabilization, cellular entry, and cell killing. CT26 cells were treated with LASNEO, and laser irradiation was applied 4 h after transfection (0.1 W cm^−2^, 2 min), followed by incubating for another 1 h in the medium. Subsequently, 2.5% glutaraldehyde was mixed with 2% paraformaldehyde. The cells were then immobilized for 1 h with the mixture solution, followed by washing with PBS, fixing with a mixture of 1% osmium acid and 1.5% potassium hexacyanoferrate, staining with uranium dioxo acetate, and dehydrating. After that, ultra‐thin cell slices were prepared and further stained with uranyl acetate and lead citrate. Finally, the slices were observed under a TEM (H‐7650, Hitachi, Japan) at 80 kV (Tsinghua University).

### ROS Generation Detection

For ROS detection, HepG2‐Luc or CT26 cells were treated with LASNEO as described above. After another 20 h incubation, cells were treated with laser irradiation and then stained with 2,7‐dichlorodihydrofluorescein diacetate (DCFH‐DA, in green) and incubated at 37 °C for 1 h, followed by CLSM and flow cytometry analysis.

### Determination of Macrophage Phenotype In Vitro

To determine the macrophage phenotype, RAW264.7 cells were seeded in a 6‐well plate and treated with IL‐4 for 48 h and then incubated with LASNEO for 4 h, followed by irradiating with 660 nm laser. After 24 h incubation, the cells were digested with trypsin and washed with PBS for three times. Then, cells were treated with FcR blocking reagent for 10 min at 4 °C. Finally, the cells were stained with CD206, CD86 antibodies and analyzed by flow cytometry.

### Animals

Male BALB/c nude mice (age 6–8 weeks) and BALB/c mice (age 6–8 weeks) for in vivo experiments were purchased from Vital River Laboratories (Beijing, China) and maintained in the Beijing Institute of Technology Laboratory Animal Center, which was a specific pathogen‐free experimental animal facility. All procedures were approved by the Institutional Animal Care and Use Committee (IACUC) of the Beijing Institute of Technology and performed under the guidelines and policies. The approval number was “BIT‐EC‐SCXK(Beijing) 2016‐0006‐M‐202016.”

### Antitumor Activity of LASNEO in HepG2‐Luc Xenograft Murine Model

HepG2‐Luc cell line xenograft tumor model was used to evaluate the anticancer effects of LASNEO. 5 × 10^6^ HepG2‐Luc cells were suspended in 1 × PBS (100 µL) and subcutaneously injected into the right axillary fossa of male BALB/c nude mice weighing ≈20 g. Mice were randomly divided into five groups with six animals per group when the tumor volume reached ≈50–100 mm^3^. Then the animals received intratumoral injections of 1) PBS, 2) NEO, 3) LANEO with laser (LANEO‐L), 4) SNEO and 5) LASNEO with laser (LASNEO‐L) every other day, respectively. LANEO were the NEO loaded with Ce6, and SNEO was the NEO loaded with siRNA. The siRNA targeting PLK1 (siPLK1) was employed in this study and dosed at 1 mg kg^−1^ in groups of SNEO and LASNEO. The Ce6 was dosed at ≈0.25 mg kg^−1^. After each injection, mice in LANEO and LASNEO groups were exposed to 660 nm laser (0.1 W cm^−2^) for 5 min. The treatment was terminated when the average tumor volume of the PBS group reached ≈1000 mm^3^. Bodyweight and tumor volume of BALB/c nude mice were recorded throughout the treatment course. The tumor volume was calculated according to the following formula: tumor volume (mm^3^) = 0.5 × length × width^2^. Bioluminescence imaging was performed at the end of the treatment course to record firefly luciferase activity expressed in HepG2‐Luc cells (IVIS Spectrum CT, PerkinElmer, Waltham, MA, US). The luciferase substrate was intraperitoneally injected into the mice, and luciferase activity was detected after 20 min. Subsequently, mice were sacrificed by cervical dislocation. Blood samples were collected and serum specimens were prepared. Ten serum biochemistry parameters including AST, ALT, ALP, ALB, TBIL, *γ*‐GT, BUN, CREA, TC, and TRIG, were then analyzed by Beijing DIAN Clinical Laboratory Co. Ltd. Moreover, a piece of tumor tissue was kept in RNA*later* solution, followed by extracting total RNA and determining the expression level of PLK1 mRNA via RT‐qPCR. Meanwhile, another piece of tumor tissue was fixed with 4% paraformaldehyde and embedded in paraffin. Tissue sections were stained with hematoxylin‐eosin (H&E) and analyzed with an inverted microscope.

### TUNEL Assay

Terminal‐deoxynucleotidyl transferase (TdT) Mediated Nick End Labeling (TUNEL) assay has been designed to detect apoptotic cells that undergo extensive DNA degradation during the late stages of apoptosis. The method was based on the ability of TdT to label blunt ends of double‐stranded DNA breaks independent of a template. Tumor were isolated at the end of the experiment and put into optimal cutting temperature compound, stored at −80 °C. Tumor frozen slices were then obtained by a frozen slicer and stained with FITC‐12‐dUTP for 1 h. After washed with PBS for three times, slices were observed by CLSM.

### Immunotherapy of LASNEO in CT26 Xenograft Murine Model

CT26 cell line xenograft tumor model was employed to evaluate the immunotherapy effect of LASNEO containing anti‐PD‐L1 siRNA (siPD‐L1). In this case, 5 × 10^6^ CT26 cells were subcutaneously injected into the right axillary fossa of male BALB/c mice weighing 16–18 g. When the tumor volume reached 80–100 mm^3^, mice were divided into five groups with ten animals per group, followed by treatment with 1) PBS, 2) NEO, 3) LANEO‐L, 4) SNEO, and 5) LASNEO‐L every other day, respectively. siRNA was dosed at 0.5 mg kg^−1^. The body weight, tumor volume, and animal survival were also recorded, and the treatment was terminated when the average tumor volume of the PBS group reached ≈2000 mm^3^. Then the tumors were isolated, weighed, and optically imaged. The organ coefficients of the liver and spleen were examined. In addition, a piece of tumor tissue was kept in RNA*later*, followed by determining the expression of PD‐L1 mRNA via RT‐qPCR. The main organs and tumors were subjected to formalin fixation, H&E staining, and microscopy observation.

### Examination of Macrophage and DC Phenotype In Vivo

According to the requirements of experimental grouping, several 6‐well plates were taken to mark the information of tumor‐bearing mice. Three milliliters of DMEM with high sugar were added to each well of the 6‐well plate, which was placed on ice until used. Tumor tissue cells were harvested and cut into pieces. Then, 1 mg mL^−1^ collagenase I and 200 µg mL^−1^ DNA enzyme I was added to tissue fragments and incubated for 30 min at 37 °C. DMEM with high glucose (containing 10% FBS) was added to each well to terminate the digestion. The tumor tissue suspension was passed through 70 µm nylon mesh to obtain a single‐cell suspension. Cells were counted and re‐suspended up to 2 × 10^7^ cells per 1 mL of PBS. FcR blocking reagent was added into cell suspension and incubated for 10 min at 4 °C. The cells were then incubated with different antibodies: CD11b, CD206, CD86 for macrophage subtypes detection, and CD11c, CD80, and CD86 for matured DC detection.

### T Cell Analysis in Tumor

Tumor tissues collected from animals in each group were digested with collagenase. The T cells were extracted with a lymphocyte separation kit (Solarbio) and incubated with FcR blocking reagent. The populations of CD4+ T, CD8+ T, and Treg cells in tumor tissues were analyzed with flow cytometry. CD3 positive T cells were initially identified and then further gated by the expressions of CD4, CD8a, and FOXP3.

### Statistical Analysis

The GraphPad Prism 8.0 was used for statistical analysis. The results were presented as mean ± SD or mean ± SEM. Student *t*‐test, One‐Way ANOVA, or Two‐Way ANOVA were employed for statistical comparison. Statistical significance was determined with 95% (*, *p* < 0.05), 99% (**, *p* < 0.01), 99.9% (***, *p* < 0.001) and 99.99% (****, *p* < 0.0001) confidence intervals.

## Conflict of Interest

Y.H. and M.Z. have filed a patent related to the study. The other authors declare no conflict of interest.

## Supporting information

Supporting Information

## Data Availability

The data that support the findings of this study are available in the supplementary material of this article.
