# Supplementary material for: Conscription of Immune Cells by Light‐Activatable Silencing NK‐Derived Exosome (LASNEO) for Synergetic Tumor Eradication
Source: Adv Sci (Weinh). 2022 Jun 4;9(22):2201135. doi: 10.1002/advs.202201135 (PMC9353410; doi:10.1002/advs.202201135)
Supplement: Supplementary file 1 — Supporting Information [file ADVS-9-2201135-s001.pdf]

ADVANCED SCIENCE
Open Access

Supporting Information

for *Adv. Sci.*, DOI 10.1002/advs.202201135

Conscription of Immune Cells by Light-Activatable Silencing NK-Derived Exosome (LASNEO) for Synergetic Tumor Eradication

*Mengjie Zhang, Wanxuan Shao, Tongren Yang, Houli Liu, Shuai Guo, Deyao Zhao, Yuhua Weng, Xing-Jie Liang and Yuanyu Huang**

Supporting Information

Light-activatable Silencing NK-derived Exosome (LASNEO) for Synergetic Tumor Eradication

*Mengjie Zhang, Wanxuan Shao, Tongren Yang, Houli Liu, Shuai Guo, Deyao Zhao, Yuhua Weng, Xing-Jie Liang, and Yuanyu Huang**

M. Zhang, W. Shao, T. Yang, H. Liu, S. Guo, Prof. Y. Weng, and Prof. Y. Huang
School of Life Science, Advanced Research Institute of Multidisciplinary Science, School of Medical Technology (Institute of Engineering Medicine); Key Laboratory of Molecular Medicine and Biotherapy; Key Laboratory of Medical Molecule Science and Pharmaceutics Engineering; Beijing Institute of Technology, Beijing 100081, China.
E-mail: yyhuang@bit.edu.cn

D. Zhao
Department of Radiation Oncology, the First Affiliated Hospital of Zhengzhou University, Erqi, Zhengzhou 450000, China.

Prof. X.J. Liang
Chinese Academy of Sciences (CAS) Center for Excellence in Nanoscience, CAS Key Laboratory for Biomedical Effects of Nanomaterials and Nanosafety, National Center for Nanoscience and Technology, Beijing 100190, China.

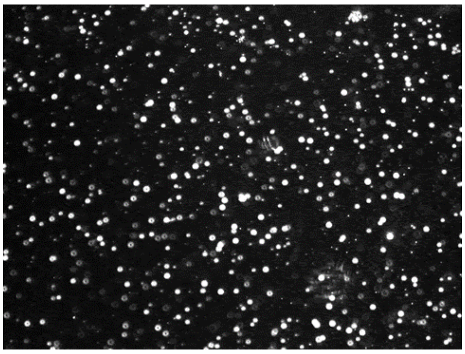

Figure S1. NTA image of NEO at peak around 120 nm in diameter and concentration of 3×10^{11} particles/mL.

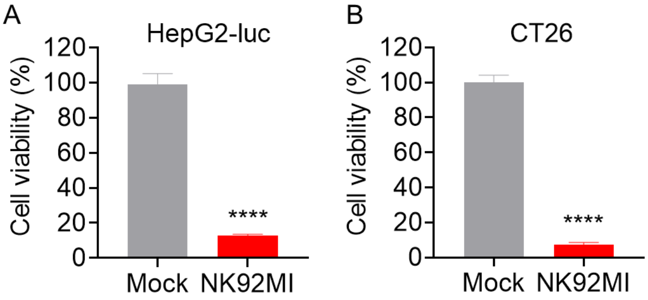

Figure S2. The cytotoxicity of NK92MI towards (A) HepG2-Luc and (B) CT26 cells. ****p < 0.0001, vs Mock.

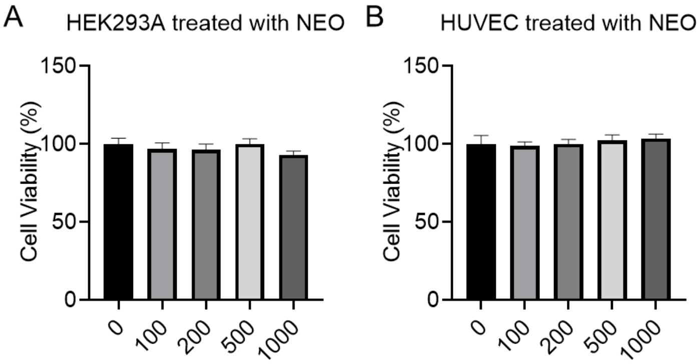

Figure S3. Cytotoxicity of NK92MI-derived exosome (NEO) evaluated in (A) HEK-293 cells and (B) human umbilical vein endothelial cells (HUVEC).

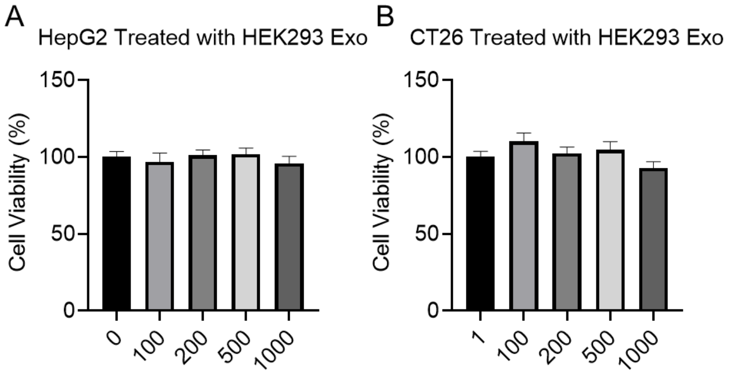

Figure S4. Cell viability of (A) HepG2 and (B) CT26 cells treated with different concentrations (ng/μL) of HEK 293-derived exosome.

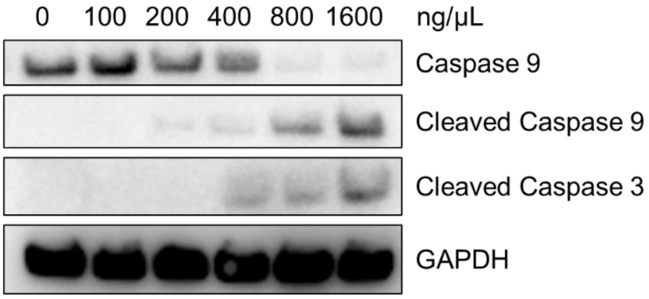

Figure S5. Western blot analysis of crucial proteins involved in apoptosis pathway. The concentrations of NEO, as determined by BCA assay, were 0, 100, 200, 400, 800, 1600 ng/μL, respectively.

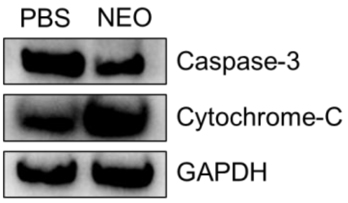

Figure S6. The crucial proteins involved in apoptosis signaling pathway in CT26 cells which treated with PBS or NEO for 24h. The finally concentration of NEO was 200 ng/μL.

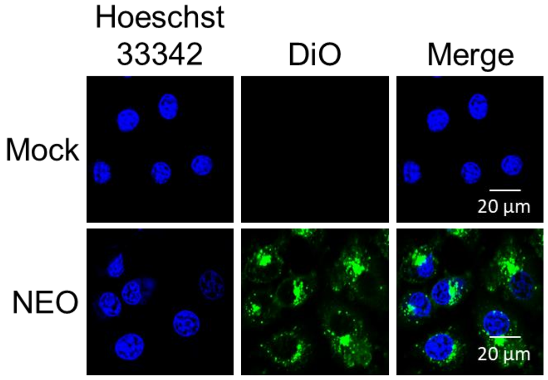

Figure S7. Confocal laser scanning microscopy (CLSM) images of HepG2-Luc cells after transfected with NEO. NEO was labeled with DiO. Scale bar, 20μm.

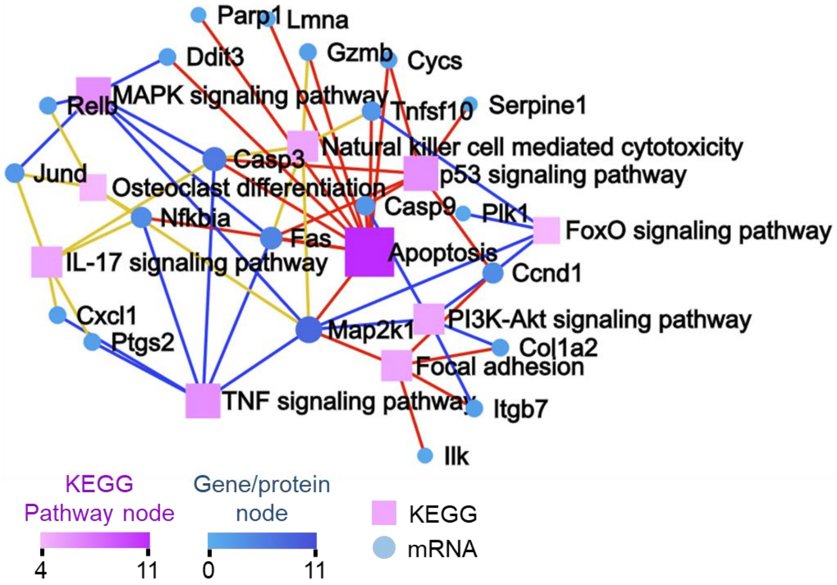

Figure S8. Genomes (KEGG) pathway enrichment analysis. RNA-seq was performed with the sample collected from NEO-treated CT26 cells, then the enrichment analysis was conducted.

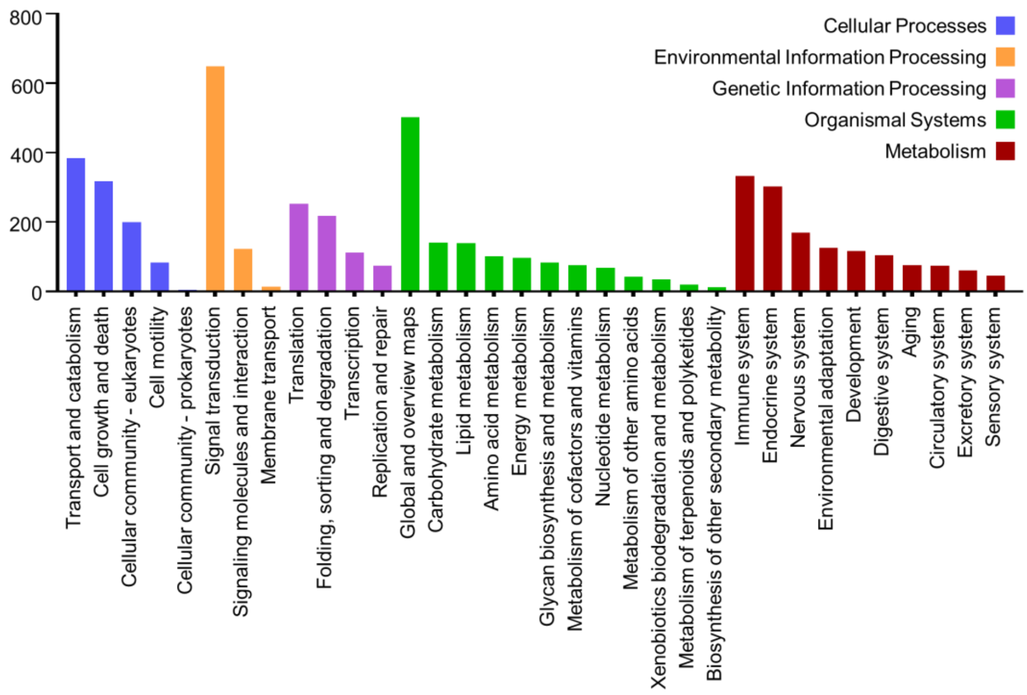

Figure S9. Kyoto Encyclopedia of Genes and Genomes (KEGG) enrichment analysis. As a result, five representative branches including cellular processes, environmental information, processing genetic information, processing metabolism and organismal systems were identified.

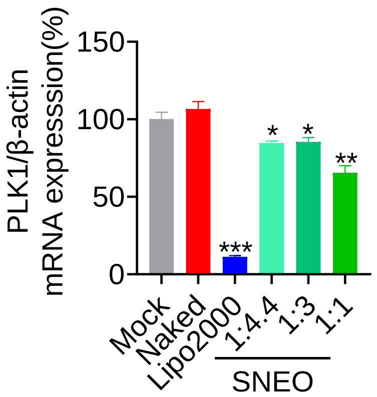

Figure S10. Relative PLK1 mRNA expression in HepG2-Luc receiving SNEO treatment at different NEO/siRNA mass ratio. NEO is short for NK cell-derived exosome, and SNEO is siRNA-loaded NEO. Naked siRNA was used as a negative control. Lipofectamine 2000 (Lipo2000) was included as a positive control. *$p < 0.05$, **$p < 0.01$, ***$p < 0.001$, vs Mock group.

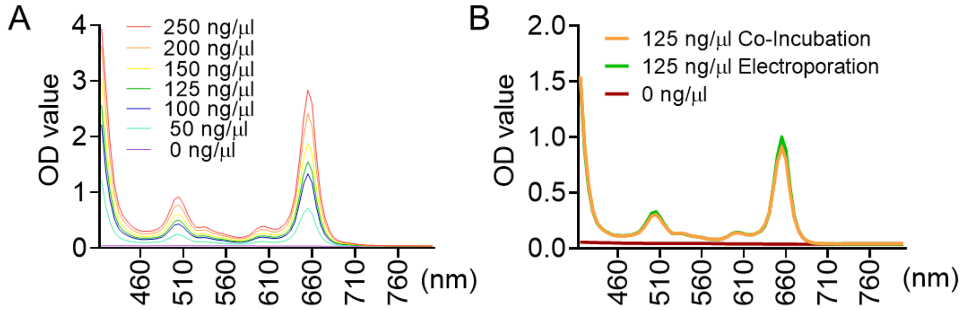

Figure S11. Determination of Ce6 loading in LASNEO that prepared by co-incubation or electroporation. (A) Standard curve of the detection of Ce6. (B) Absorption signal of Ce6 loaded in LASNEO.

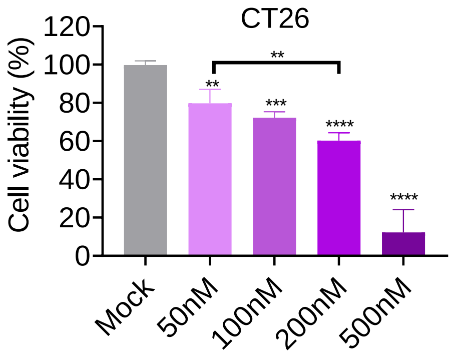

Figure S12. Cancer cell killing capability of LASNEO recorded in CT26 cells. The cells were treated with LASNEO and irradiated with 660 nm laser for 2 min. $**p < 0.01$, $***p < 0.001$ and $****p < 0.0001$.

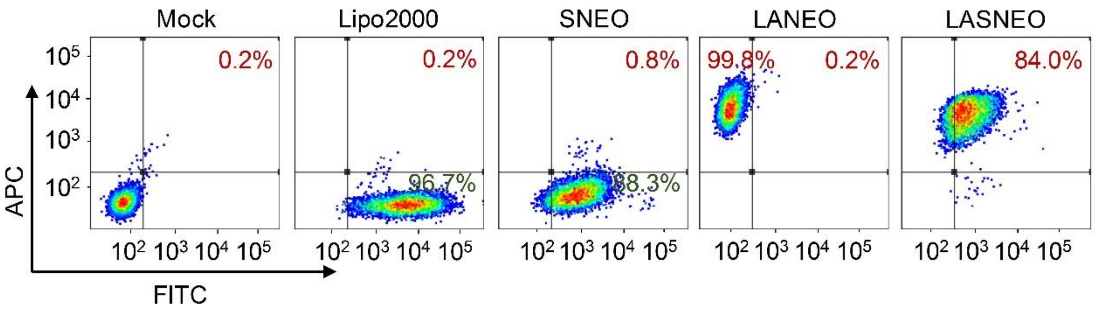

Figure S13. Cellular uptake efficiencies of SNEO, LANEO, LASNEO and Lipo2000 analyzed by flow cytometry in HepG2-Luc cells. FAM-labeled siRNA (in green) was employed in this assay. Fluorescence signal of FAM and Ce6 were recorded. Lipo2000 incubated with FAM-labeled siRNA was used as a positive control.

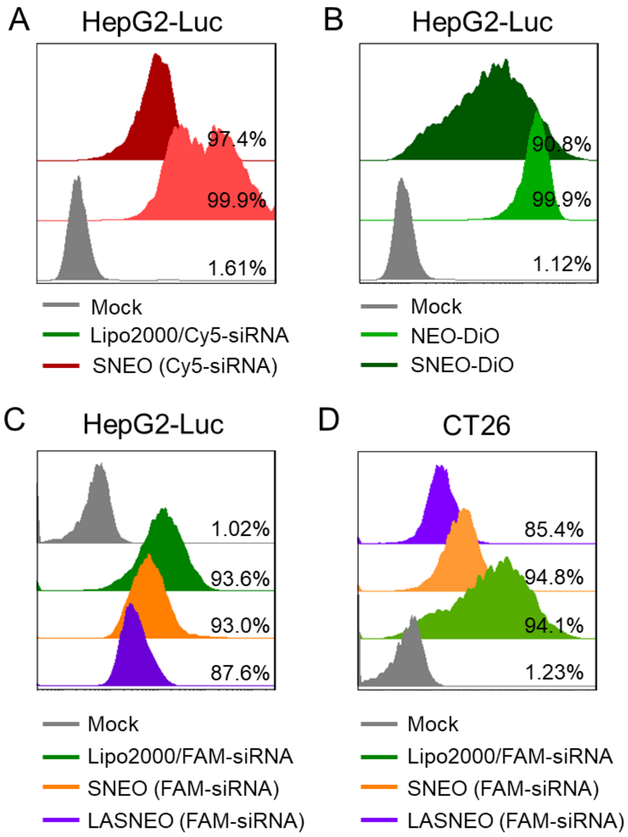

Figure S14. Cellular uptake efficiencies of NEO, SNEO, LASNEO and Lipo2000 analyzed by flow cytometry. Lipo2000 incubated with Cy5 or FAM siRNA was used as a positive control. (A-B) siRNA was labeled with Cy5 and NEO was marked with DiO. (C-D) siRNA was labeled with FAM.

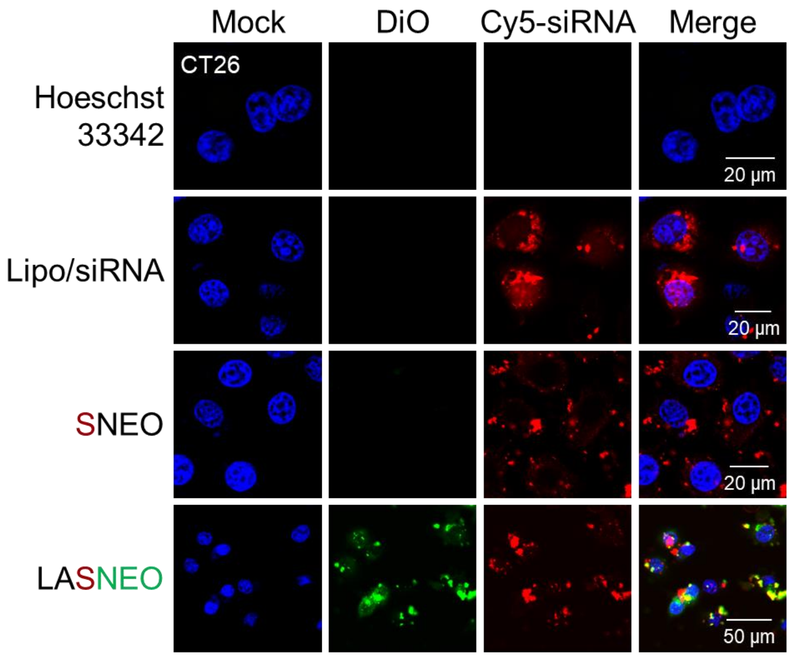

Figure S15. Confocal observations of internalization of SNEO and LASNEO in CT26 cells. siRNA was labeled with Cy5 (in red). Nuclei and exosome were stained with Hoechst 33342 (in blue) and DiO (in green), respectively.

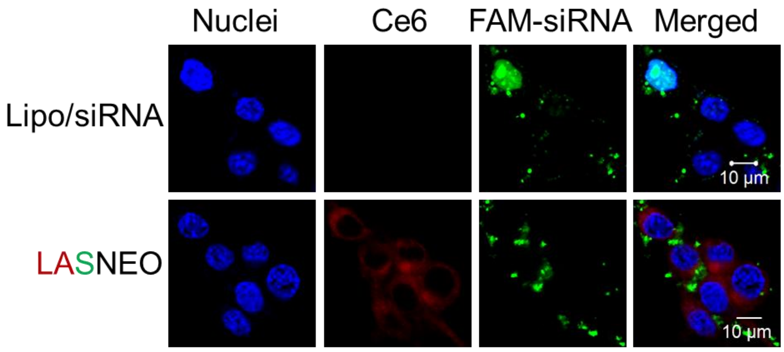

Figure S16. Confocal observations of LASNEO internalization in CT26 cells. Here, Ce6 (in red) was analyzed. Nuclei and NEO were stained with Hoechst 33342 (in blue) and DiO (in green), respectively.

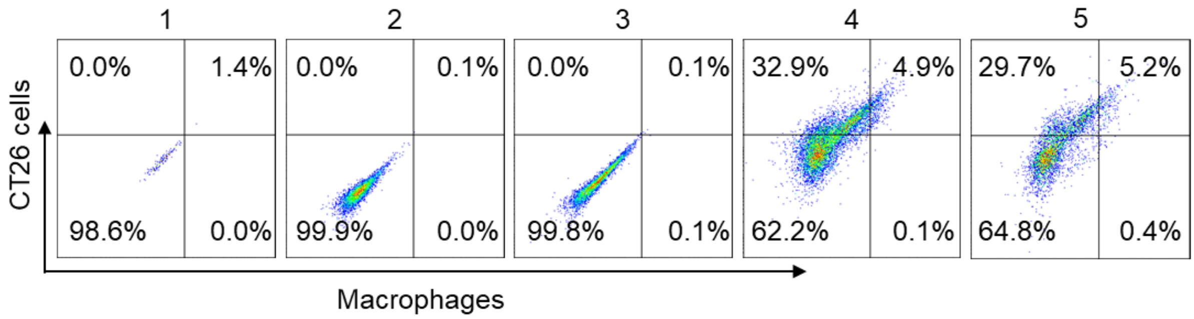

Figure S17. FACS-recorded the ratios of cellular uptake in macrophages and CT26 cells. Macrophages and CT26 cells were co-cultured, and LASNEO were labeled with DiO in this assay. Co-cultured cells treated with (1) PBS; (2) LASNEO; (3) LASNEO and sorted with isotype control (F4/80 and SDCCAG3) antibody; (4) LASNEO loaded with PD-L1 siRNA and sorted with F4/80 and SDCCAG3 antibody; (5) LASNEO loaded with siNC and then sorted with F4/80 and SDCCAG3 antibody, respectively.

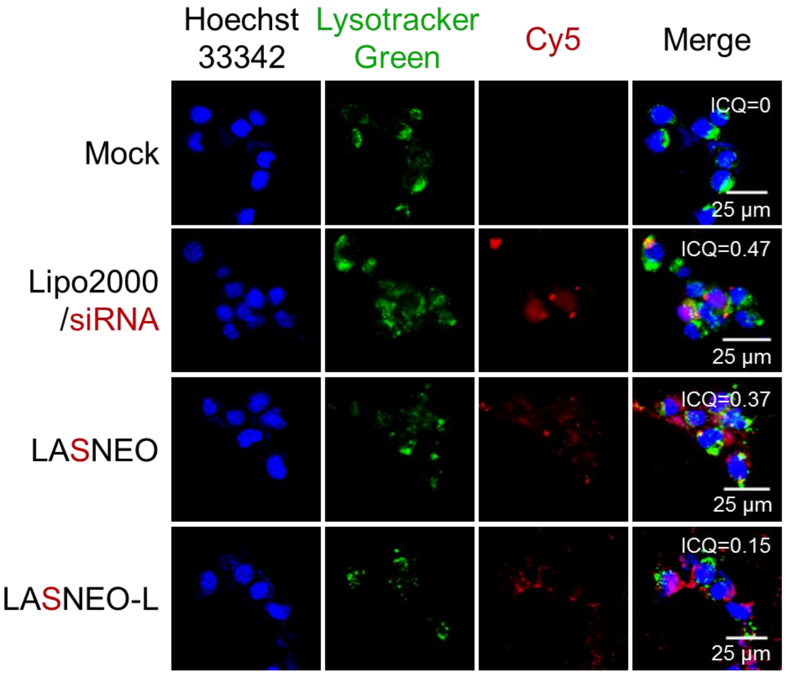

Figure S18. Confocal analysis of siRNA subcellular localization in CT26 cells. The cells were treated with LASNEO with or without irradiation at 4 hours after transfection. Here, siRNA was labeled with Cy5 (in red). Endosome/lysosome and nuclei were stained with Lysotracker Green (in green) and Hoechst 33342 (in blue), respectively. Intensity correlation quotient (ICQ) analysis was performed to determine the colocalization. Scale bar: 25 μm.

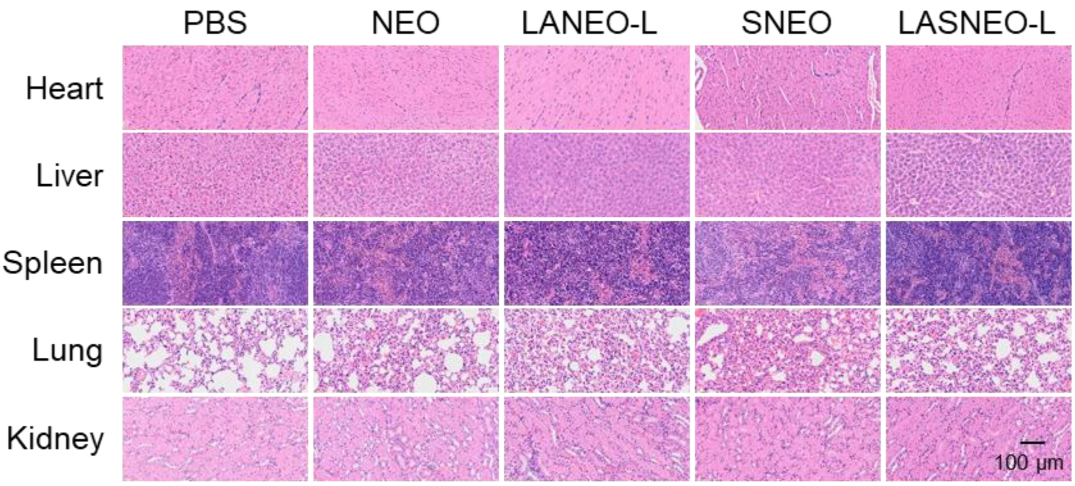

Figure S19. H&E staining of the main tissues collected from the animals receiving indicated treatments in HepG2-Luc cell xenograft tumor model. Scale bar, 100 μm.

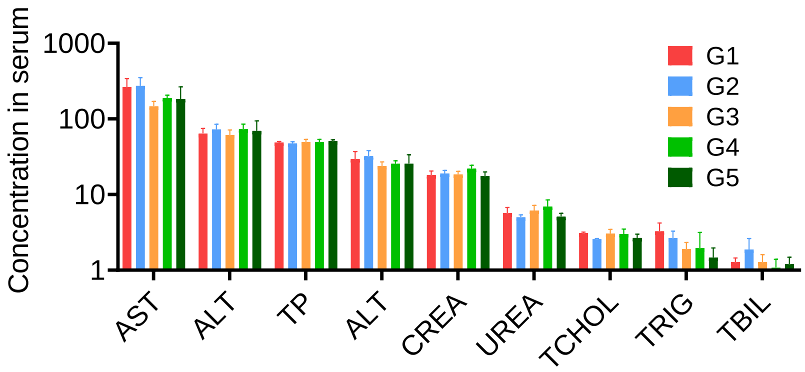

Figure S20. Serum biochemistry parameters recorded at the end of experiment in CT26 xenograft tumor model. Nine indicators were analyzed, which included including aspartate aminotransferase (AST, U/L), alkaline phosphatase (ALP, U/L), total protein (TP, g/L), alanine aminotransferase (ALT, U/L), serum creatinine (CREA, μmol/L), blood urea nitrogen (BUN or UREA, mg/dL), triglyceride (TRIG, mmol/L), total bilirubin (TBIL, μmol/L).

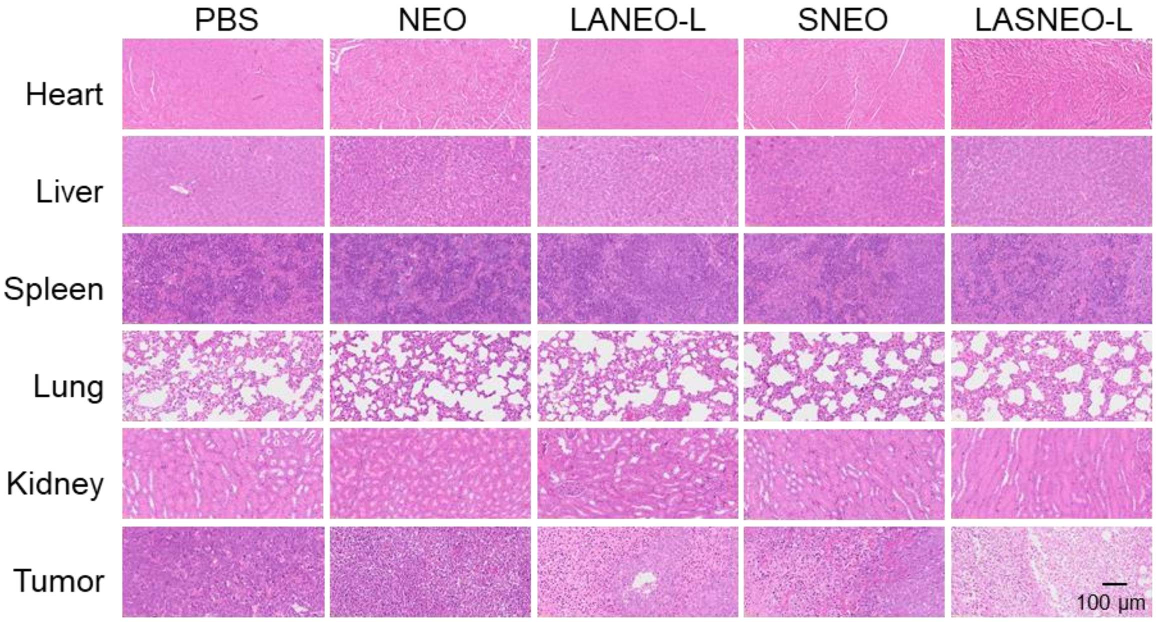

Figure S21. H&E staining of the main tissues collected from the animals receiving indicated treatments in CT26 cell xenograft tumor model. Significant apoptosis was observed in tumor section in LASNEO-L group. Scale bar, 100 μm.
